# Barefoot walking is more stable in the gait of balance recovery in older adults

**DOI:** 10.1186/s12877-022-03628-w

**Published:** 2022-11-25

**Authors:** Xiping Ren, Maeruan Kebbach, Sven Bruhn, Qining Yang, Huijie Lin, Rainer Bader, Thomas Tischer, Christoph Lutter

**Affiliations:** 1 College of Physical Education and Health Sciences, Zhejiang Normal University, 688 Yingbin Road, Jinhua, 321000 China; 2 Department of Orthopedics, Biomechanics and Implant Technology Research Laboratory, Rostock University Medical Center, Doberaner Strasse 142, 18057 Rostock, Germany; 3 Institute of Sport Science, University of Rostock, 18051 Rostock, Germany; 4 Department of Joint Surgery, The Affiliated Jinhua Hospital, Zhejiang University School of Medicine, Jinhua, 321099 China; 5 School of Physical Education, Taizhou University, Linhai, 318000 China

**Keywords:** Falls, Aged, Coefficient of variation, Spatiotemporal parameters, Footwear

## Abstract

**Background:**

Perturbation-based balance training on a treadmill is an emerging method of gait stability training with a characteristic task nature that has had positive and sustained effects on balance recovery strategies and fall reduction. Little is known about the effects produced by shod and barefoot walking. We aimed to investigate which is more appropriate, shod or barefoot walking, for perturbation-based balance training in older adults.

**Methods:**

Fourteen healthy older adults (age: 68.29 ± 3.41 years; body height: 1.76 ± 0.10 m; body mass: 81.14 ± 14.52 kg) performed normal and trip-like perturbed walking trials, shod and barefoot, on a treadmill of the Gait Real-time Analysis Interactive Lab. The marker trajectories data were processed by Human Body Model software embedded in the Gait Offline Analysis Tool. The outcomes of stride length variability, stride time variability, step width variability, and swing time variability were computed and statistically analyzed by a two-way repeated-measures analysis of variance (ANOVA) based on gait pattern (normal gait versus perturbed recovery gait) and footwear condition (shod versus barefoot).

**Results:**

Footwear condition effect (*p* = 0.0310) and gait pattern by footwear condition interaction effect (*p* = 0.0055) were only observed in swing time variability. Gait pattern effects were detected in all four outcomes of gait variability.

**Conclusions:**

Swing time variability, independent of gait speed, could be a valid indicator to differentiate between footwear conditions. The lower swing time variability in perturbed recovery gait suggests that barefoot walking may be superior to shod walking for perturbation-based balance training in older adults.

## Background

Falls during walking put older adults at risk of serious injuries, e.g., fractures, brain injuries, or even death [1–3]. Trips are the most prevalent known cause of outdoor falls [4] and are considered to be the most challenging of falls [5, 6].

Due to the task-specific nature of improving gait stability in older adults at risk of falls in daily life [3, 7], perturbation-based balance training has been applied to simulate the occurrence of falls in a real environment [8, 9]. This form of training has produced positive and sustained effects on balance recovery strategies and on reducing falls in older adults and those with gait disorders [8, 10]. However, to our knowledge, researchers have not yet fully explored footwear condition (shod versus barefoot) as an independent variable that may affect the training effect. To date, clear evidence on whether shod or barefoot walking is preferable in perturbation-based balance training is still pending.

Footwear has a direct effect on gait performance [11–13], which has led to the popularity of barefoot locomotion (walking and running) in recent years and has evoked an increasing scientific interest in its benefits and limitations [14]. Typically, a well-fitting standard shoe with laces, a low and wide heel, a firm heel collar, and a grooved, moderately hard sole is recommended for older adults in rehabilitation and daily use [11]. However, footwear interferes with balance and, as a result, the risk of slipping, tripping, and falls by varying somatosensory feedback to the foot and ankle and altering frictional conditions at the shoe/floor interface [15], which has been demonstrated to hinder kinesthesia [16]. Due to the structural limitations of the shoe, forefoot spreading under load can be reduced [13]. As the sole thickness increases, neutral running footwear can significantly increase the activation of the peroneus longus muscle and interfere more with ankle stability [17]. An elevated heel of only 4.5 cm height significantly impairs balance in older adults whereas a high heel collar and a hard sole showed trends towards being beneficial [18]. Minimalist shoes improve dynamic stability in older adults better than barefoot [19], while it remains to be investigated whether it is superior to conventional footwear [20]. A recent study highlights barefoot walking has clinical potential based on gait stability and variability outcomes in both young and older adults [12]. Compared to normal gait, perturbated gait is more challenging and the essence lies in stability control. It has been suggested that gait variability, i.e., the coefficient of variation or standard deviation of spatiotemporal parameters, may better reflect subtle changes in natural gait fluctuations [21, 22], as it is a unique indicator of walking control [23] and maybe a direct predictor of falls in older adults [24–27]. The smaller the gait variability, the higher the gait stability [24, 27]. An increase in gait variability is also a clinically relevant biomarker for fall risk diagnosis [28, 29].

Therefore, we aimed to investigate whether gait variability parameters associated with falls remain consistent in healthy older adults during perturbation-based balance training while shod and barefoot walking to determine which footwear condition is more appropriate. We proposed the following hypothesis that barefoot walking could be more stable in both normal and perturbed recovery gait.

## Methods

### Participants

A total of 14 community-dwelling healthy older adults (age: 68.29 ± 3.41 years; body height: 1.76 ± 0.10 m; body mass: 81.14 ± 14.52 kg; Body Mass Index, BMI: 26.18 ± 5.00 kg/m^2^; Timed Up and Go test, TUG: 10.21 ± 1.21 s; 4 females) were recruited to participate in this study. The 14 sample sizes were selected based on a priori statistical power analysis obtained with G*Power v3.1.9.7, using a two-factor repeated measures analysis of variance (ANOVA) within interaction (α = 0.05, 1-β = 0.80, effect size f = 0.50, correlation among repeated measures = 0.50). The inclusion criteria for participants were no history of lower extremity disorders or injuries as well as no foot problems and gait abnormalities like spastic, scissors, steppage, waddling, and propulsive gait. The dominant leg was right. Participants were asked to wear tight-fitting athletic shorts and T-shirts provided by the investigation, as well as their own appropriate neutral running shoes. Written consent was obtained after comprehensive information before the test was conducted. Ethical approval was granted by the committee of the Rostock University Medical Center, Germany (A2019-0231). The Declaration of Helsinki was followed in all measurements.

### Measurement protocol

**Fig. 1:**
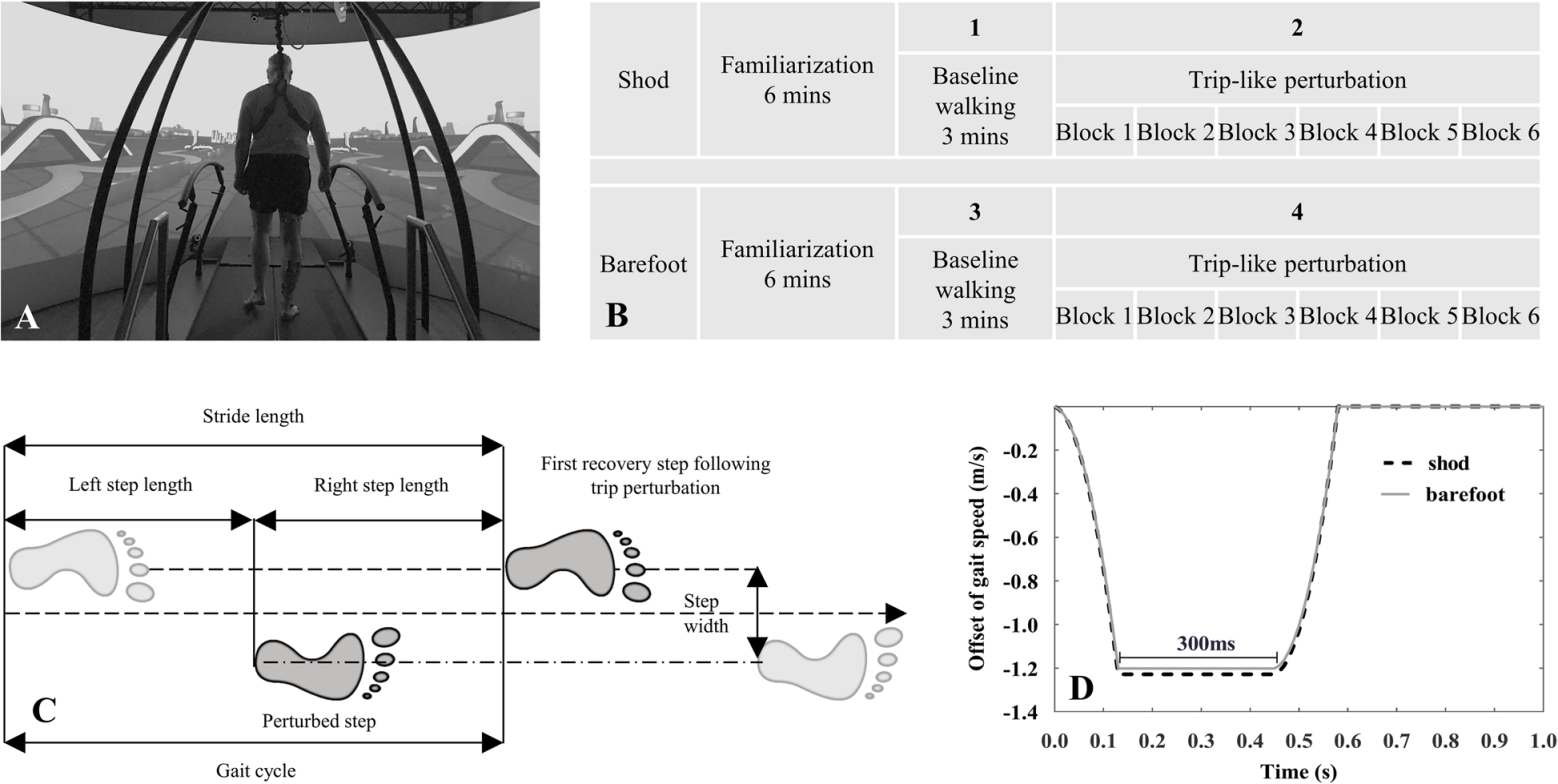
Investigation scenario for this study using the Gait Real-time Analysis Interactive Lab (**A**); Experimental protocol of the trip-like perturbation training: the protocol consisted of two sessions of shod and barefoot walking, for a total of four trials. Each session started with a 6-min familiarization during which the participant’s self-selected speed was recorded. The first trial was performed by walking shod at a self-selected speed for 3 min, and the second trial was performed with 6 blocks of trip perturbation with an interval of 15–20 s between each. The third and fourth trials of barefoot walking were performed with the exact process as shod walking (**B**); Schematic diagram of spatiotemporal parameters of the gait cycle, trip perturbation occurrence step, and the first recovery step (**C**); Trip perturbation is where the belt decelerates at 3 m/s.^2^ at normal gait speed until the speed is reduced by almost 1.2 m/s and held for 300 ms, and then accelerates to normal gait speed. Schematic diagrams are the curves generated for participant with an average gait speed of 1.35 m/s (shod, black dashed line) and 1.32 m/s (barefoot, gray solid line) with the x and y axes representing the time and speed offset, respectively (**D**)

The Gait Real-time Analysis Interactive Lab (GRAIL, Motek Medical B.V., Houten, the Netherlands), with an integrated 10-camera motion capture system (Vicon Metrics Ltd., Oxford, United Kingdom), was utilized to capture the three-dimensional marker trajectories in a virtual reality environment at a sampling frequency of 100 Hz (Fig. 1A). The D-Flow software v3.34 (Motek Medical B.V., Houten, the Netherlands) was used for signal triggering and data acquisition. According to the Human Body Model 2 (HBM2, Motek Medical B.V., Houten, the Netherlands), 26 reflective markers were attached to the anatomical bone landmarks. A total of four trials were performed for each participant, including normal and trip-induced walking with shod and barefoot condition, respectively (Fig. 1B). Thus, gait patterns included both normal and perturbed gait as well as footwear conditions including shod and barefoot walking in this study. An adequate break (approximately 5 min) was given between each trial until the participant felt ready to continue with the next test [19]. A safety rope system was used throughout the test to prevent potential falls of participants.

A familiarization period of 6 min was maintained before the formal measurements in shod and barefoot, respectively [30–32]. During this period, the average of the participant's two self-reported comfortable speeds was used as normal speed [6]. After the self-selected speed was determined, normal walking was performed for three minutes each and trip-induced perturbed walking was performed six times each in the subsequent four trials. The timing of the occurrence of the perturbations was pseudo-random, with intervals of 15–20 s. The novel trip-like perturbation induced by treadmill belt deceleration was automatically delivered by a custom-made program written in the Lua language based on D-Flow software with an intensity of 3 m/s^2^. The perturbation occurred at the right heel strike (Fig. 1C) and lasted for 300 ms, then returned to normal speed (Fig. 1D), as described in our latest study [33].

### Data processing

The data involved were processed using the HBM software embedded in the Gait Offline Analysis Tool (GOAT v4.1, Motek Medical, B.V., the Netherlands). The three-dimensional coordinates of the markers were filtered with a second-order low pass Butterworth (zero-phase) at a 6 Hz cutoff frequency [34, 35]. The local maxima in the anterior–posterior position of the heel marker relative to the pelvis was used to identify the heel-strike and toe-off events [36]. The gait cycle is defined as shown in Fig. 1. The following outcome gait parameters were identified via the data extraction process: stride length, stride time, step width, and swing time. Gait variability was defined as the coefficient of variation (CoV) calculated as the standard deviation (SD) divided by the mean value [25, 37].

The first recovery step (Rec1) following perturbation is the most important protective strategy [38, 39], where humans can quickly regain stability and maintain balance from the same type of perturbation [40]. To eliminate the unknown anticipation of the body proprioceptive response to the first perturbation, the Rec1 of the 2nd-6th perturbation was processed and averaged. For normal walking, 25 consecutive strides were averaged for each participant [28, 41].

### Statistical analysis

GraphPad Prism v9.3.1 (GraphPad Software Inc., San Diego, CA, USA) was utilized for all statistical analysis. Normality was checked for each variable using the D’Agostino-Pearson test. The Wilcoxon signed rank test and paired t-test were performed to analyze the statistical differences in normal gait speed and perturbed recovery gait speed in shod and barefoot walking, respectively. A two-way repeated-measures ANOVA with gait pattern and footwear condition as factors, based on a General Linear Model, was applied to identify differences in gait variability [19]. When a significant main effect or factor interaction effect was found, a Tukey’s post hoc analysis with individual variances was performed to correct for multiple comparison tests. Each *p* value was adjusted to account for any pairwise comparisons. The significance level was set at *p* < 0.05.

## Results

Gait speeds for normal gait and the first recovery step following trip perturbation when walking shod and barefoot are presented in Table 1. There was a significant decrease in barefoot walking speed compared to shod walking in both normal (*p* = 0.0103) and trip-Rec1 gaits (*p* < 0.0001).

**Table 1:** Gait speed for barefoot and shod walking in different gait pattern. The gait speed for Normal is the mean value of 25 consecutive strides, and the gait speed for trip-Rec1 is the mean value of the first recovery step following the 2nd-6th trip perturbation

Variable	Gait pattern	Footwear condition	Mean ± standard deviation (SD)	*p*-value
Gait speed (m/s)	Normal	Shod	1.35 ± 0.13	0.0103
		Barefoot	1.32 ± 0.14	
	Trip-Rec1	Shod	1.29 ± 0.16	< 0.0001
		Barefoot	1.23 ± 0.16	

**Fig. 2:**
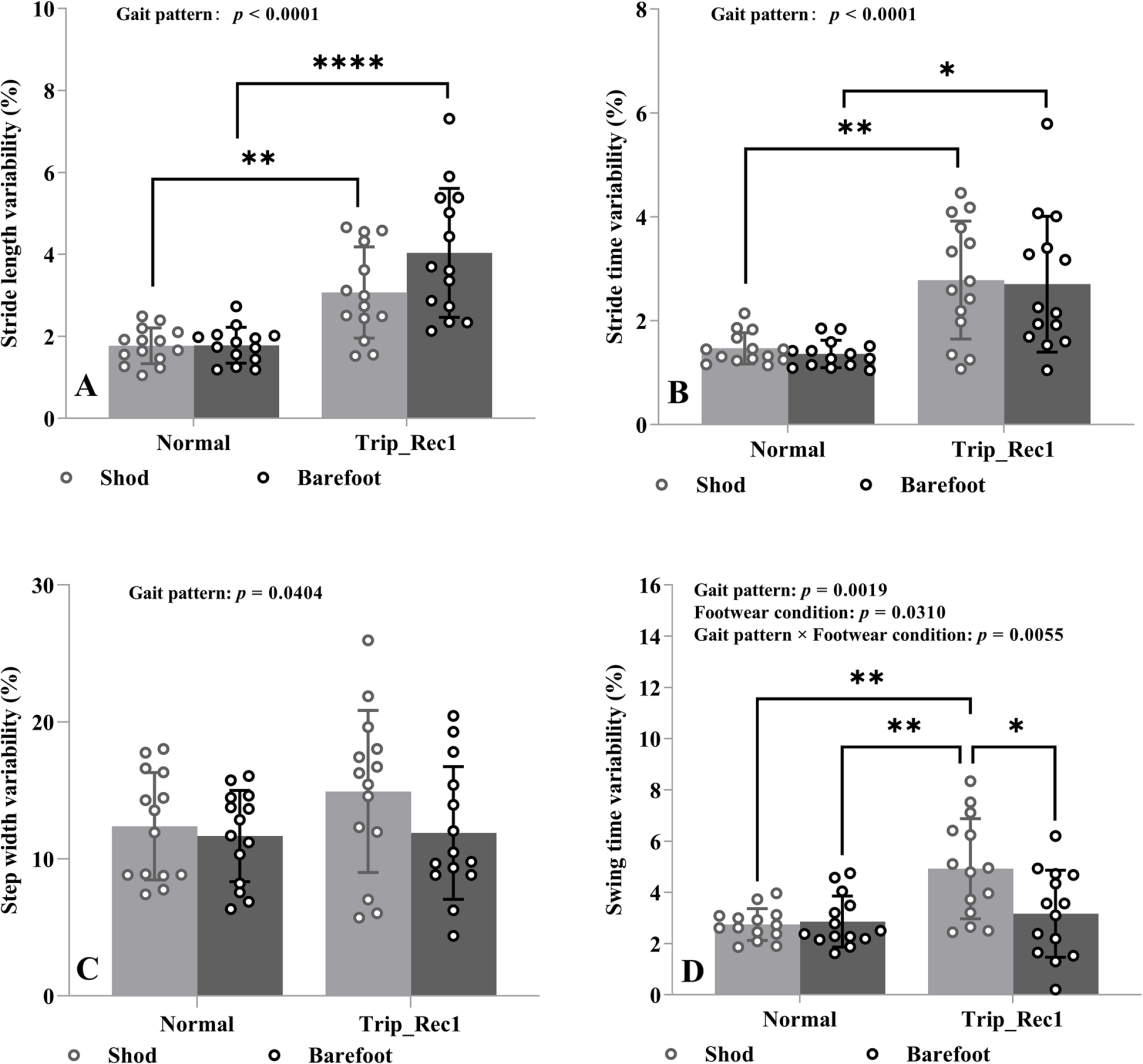
Effects of gait pattern (normal versus trip-Rec1) and footwear condition (shod versus barefoot walking) on stride length variability (**A**), stride time variability (**B**), step width variability (**C**), and swing time variability (**D**). The interleaved scatter with bars and error bars indicates mean values and standard deviation, showing with the individual values. Variability was defined as the coefficient of variation (CoV, %) calculated as the standard deviation divided by the mean value. Gait pattern effect, footwear condition effect and the interaction effect between the two are shown at the top of each plot. Asterisks represent p-value classification for Tukey’s post hoc multiple comparisons. *: *p* < 0.05, **: *p* < 0.01; ****: *p* < 0.0001

The effects of gait pattern and footwear condition on stride length variability, stride time variability, step width variability, and swing time variability in older adults are demonstrated in Fig. 2.

The results of two-way repeated measures ANOVA revealed significant gait pattern effects in terms of the outcomes of stride length variability (F_(1,13)_ = 51.24, *p* < 0.0001, η^2^_p_ = 0.4253, Fig. 2a), stride time variability (F_(1,13)_ = 32.47, *p* < 0.0001, η^2^_p_ = 0.3752, Fig. 2B), step width variability (F_(1,13)_ = 5.180, *p* = 0.0404, η^2^_p_ = 0.0223, Fig. 2C), and swing time variability (F_(1,13)_ = 14.94, *p* = 0.0019, η^2^_p_ = 0.1456, Fig. 2D). Tukey’s post hoc tests for multiple comparisons revealed that increased stride length and stride time variability both in shod and barefoot walking, and increased swing time variability in shod walking were observed in trip_Rec1 relative to normal gait (Fig. 2A–D). This indicates that older adults have an increased likelihood of falling during trip_Rec1 compared to normal gait. However, no significant differences for step width were found in Tukey’s post hoc multiple comparisons (Fig. 2C).

No significant footwear condition effects and gait pattern by footwear condition interaction effects were found for stride length variability (F_(1,13)_ = 3.978, *p* = 0.0675, η^2^_p_ = 0.0324; F_(1,13)_ = 4.143, *p* = 0.0627, η^2^_p_ = 0.0306, Fig. 2A), stride time variability (F_(1,13)_ = 0.1250, *p* = 0.7293, η^2^_p_ = 0.1790; F_(1,13)_ = 0.0033, *p* = 0.9551, η^2^_p_ = 0.00004, Fig. 2B), or step width variability (F_(1,13)_ = 0.2.431, *p* = 0.1430, η^2^_p_ = 0.0411; F_(1,13)_ = 0.9052, *p* = 0.3587, η^2^_p_ = 0.0156, Fig. 2C).

Significant footwear condition effect as well as gait pattern by footwear condition interaction effect were only found in swing time variability (F_(1,13)_ = 5.852, *p* = 0.031, η^2^_p_ = 0.0632 and F_(1,13)_ = 11.06, *p* = 0.006, η^2^_p_ = 0.0829, Fig. 2D). Tukey’s post hoc analysis revealed significant differences in swing time variability between shod walking and barefoot walking in trip_Rec1 gait (Fig. 2D). Specifically, lower swing time variability (better) in barefoot walking in trip_Rec1 gait was observed.

## Discussion

We examined stride length variability, stride time variability, step width variability, and swing time variability, between shod and barefoot walking in normal and trip-Rec1 gait among older adults during perturbation-based balance training. The main findings of the present study were, that stride length, stride time, and step width variability all had gait pattern main effects. Swing time variability had both gait pattern, footwear condition main effects and gait pattern by footwear condition interaction effects.

Significant differences in stride length, stride time, step width and swing time variability were observed across normal gait versus trip_Rec1 gait. Variability of spatiotemporal parameters provides a feasible approach for a quantitative assessment of gait stability [42]. A larger relative level of variability was indicated by a higher CoV [25, 43]. Significantly increased gait variability, as a response to perturbations, might be associated with poorer stability of the recovery step following a perturbation [25, 44]. To some extent, it indicated that the trip perturbation setup in this study was sufficient to cause an unstable recovery gait and even induce the risk of falls. Although no real cases of falls occurred in any of the participants during the investigation, these results seem to demonstrate that even minor external perturbation magnitudes can elicit stepping reactions in older adults and these responses are highly comparable to responses elicited by larger perturbation magnitudes [45], leading to a significant increase in gait variability of spatiotemporal parameters. These results provided a prerequisite for demonstrating the proposed hypothesis, i.e., whether the footwear condition of shod and barefoot walking induced gait variability in the same gait patterns of normal or trip-Rec1 gait.

In normal gait, barefoot walking could reduce the risk of falling [12] according to gait variability measurements [46]. However, no differences were observed in stride length variability, stride time variability, and step width variability between shod and barefoot walking either in normal or trip_Rec1 gaits in the present study. Footwear condition differed significantly only in swing time variability of trip_Rec1 gait as well as footwear condition by gait pattern, showing a smaller CoV in the case of barefoot walking.

Higher stride length variability is an important indicator of poor balance mechanisms, indicating the body’s inability to improve or recover from future fall-related events [47]. Irrespective of single-task, motor dual-task, or cognitive dual-task, barefoot walking results in a significantly slower gait pattern accompanied by increased stride length variability compared to standard shod walking [11]. These researchers suggested that barefoot walking is not recommended for older women since it could have a detrimental effect on gait patterns [11]. Separately, a recent study on barefoot and minimalist shoe walking showed a significant effect of stride length variability in both young and older adults with footwear condition. Stride length variability was significantly lower in the minimalist shoe condition [19]. The effect of footwear on stride length was not equivalent [48]. Our findings showed no statistical difference in stride length variability between shod and barefoot walking. Both of the above-mentioned studies were conducted overground, while our study was performed on a treadmill. Two recent systematic reviews indicate that saptiotemporal outcomes for treadmill and overground locomotion (walking and running) are essentially equivalent [49, 50], therefore, further studies are still required to confirm the equivalence of the stride length variability when walking on different conditions.

An increase in stride time variability indicates that the gait pattern is less rhythmic [51]. Stride time variability in our study was not consistent with a recent study [12], from which it is known that stride time variability in older adults when overground barefoot walking is lower than in those shod walking. It has been suggested that stride time variability increases with decreasing gait speed [28]. However, some studies concluded that there is no negative correlation between the two [52]. Our data were consistent with this study. Barefoot walking speed decreased significantly compared to shod walking for both normal and recovery gait, but there was no significant difference in stride time variability. Stride time variability may be the result of a multifactorial interaction, such as muscle strength, balance function, and gait speed [25]. Nevertheless, the relationship with footwear conditions has not been determined. From our data, we suspect that gait training on a treadmill with or without shoes has no direct effect on this metric.

Step width variability represents the more sensitive descriptor of locomotion control of older and young adults [53, 54]. As an indicator of the required active control [55], inaccurate control is likely to lead to increased step width variability in older adults [54]. The criteria for the step width variability were classified as: low, < 7–8%; medium, 8–27%; and high, > 27–30% according to Skiadopoulos et al. [54]. Older adults with extreme step width variability have a higher frequency of falls, specifically explained by the fact that individuals with too low step width variability may lack the necessary skills to adjust their step width to maintain balance, while too high step width variability indicates unsteady walking in clinical practice [56]. Our results are in the middle range in both shod and barefoot walking. It has been reported that footwear use is an influential factor in gait variability in healthy older adults, and habitual footwear increases step width variability [57]. Our findings were inconsistent with those described above. No statistical differences were found for step width variability between footwear condition for either normal or trip-Rec1. Although the participants were all similarly aged older adults, gait speed was not consistent. One study reported that step width variability was associated with falls in older adults walking at near-normal speeds, but not in those with gait speeds below 1.0 m/s [56]. Whether the inconsistency in results was related to gait speed requires further investigation.

Swing time variability was the only one of the four variables of gait variability we investigated that had both the main effect of footwear condition and interaction effect between footwear condition by gait pattern. This is not consistent with some of the results of a previous study. Grabiner et al. [58] reported that swing time variability was typically affected by disease and degree of aging, but does not appear to be affected by the subject’s age, gait speed, or the presence of shoes. Another controlled study of Parkinson patients versus a healthy population confirmed the finding that swing time variability was not affected by gait speed [23], which could be a speed-independent predictor of stability and fall risk [23]. Swing time variability may be mainly determined by the balance control mechanism [59]. If so, we could speculate that swing time variability on treadmill gait perturbation measurements may be a useful indicator to explore gait stability control mechanisms under conditions of constant, controlled or variable gait speed, as well as to gain insight into whether it is influenced by other external factors, such as footwear condition, perturbation intensity, etc. Our hypothesis was also partially confirmed, namely, that swing time variability could be an important dependent variable in differentiating footwear condition during perturbation-based balance training on a treadmill.

Our study is not without limitations. Firstly, our findings may only be generalizable to a non-habitually barefoot older population. The different foot-strike patterns of habitual barefoot and non-habitual barefoot may lead to differences in the spatiotemporal parameters of gait [13]. However, this seems to be of only minor importance for the question of whether training is better with shoes or without. In the present experimental design, all participants wore well-fitting neutral running shoes. A monolithic form of footwear was compared to walking barefoot, which may limit the applicability of the findings to diverse footwear [12]. Gait variability was reduced when walking in minimalist shoes compared to walking barefoot, with equivalent age group effects [19]. Moreover, different footwear has differential sole thickness and hardness, which may affect the balance of older adults [60]. Future studies will be necessary to include multifunctional footwear or socks-only versus barefoot conditions depending on the specific study population, such as patients with foot injuries or gait abnormalities, to differentiate the applicability of footwear condition for perturbation-based balance training on a treadmill. Secondly, the number of first recovery steps after perturbation included in the study was relatively small. However, to date, the number of steps/strides required to reach reliability of gait variability is still under exploration [61]. Moreover, the number of steps required varies for different indicators of spatiotemporal parameter variability [62], ranging from less than six to more than several hundred steps [28, 62–64]. It has been revealed that a single familiarization trial can significantly improve the reliability of gait variability in older adults [31, 65], which could take as little as 6 min on the treadmill [28, 32]. Last but not least, “our experimental design was to apply perturbation-based balance training to simulate the likelihood of a fall occurring” does not seem to hold, as no falls were observed. Further studies on trip perturbations leading to falls are needed to confirm our findings.

## Conclusion

Swing time variability, independent of gait speed, could be a valid indicator to differentiate between footwear condition. The lower swing time variability in perturbed recovery gait suggests that barefoot walking might be superior to shod walking for perturbation-based balance training in older adults. Whether in a clinical setting or in biomechanics, swing time variability based on spatiotemporal parameters is an easy-to-measure, easy-to-assess approach that is likely to have promising applications as an assessment of footwear conditions in various populations at risk of falls to evaluate the effects of perturbation-based balance training.

## Data Availability

The datasets generated during the current study are available from the corresponding author upon reasonable request.

## Abbreviations

CoV: Coefficient of variation
Rec1: The first recovery step
Trip_Rec1: The first recovery step following trip perturbation
ANOVA: Analysis of variance
SD: Standard deviation
GOAT: Gait Offline Analysis Tool
HBM: Human Body Model
GRAIL: Gait Real-time Analysis Interactive Lab
